# Effectiveness of tobacco control television advertisements with different types of emotional content on tobacco use in England, 2004–2010

**DOI:** 10.1136/tobaccocontrol-2013-051454

**Published:** 2014-07-18

**Authors:** M Sims, T Langley, S Lewis, S Richardson, L Szatkowski, A McNeill, A B Gilmore

**Affiliations:** 1 Department for Health, UK Centre for Tobacco and Alcohol Studies, University of Bath, Bath, UK; 2 Division of Epidemiology and Public Health, UK Centre for Tobacco and Alcohol Studies, University of Nottingham, Nottingham City Hospital, Nottingham, UK; 3 UK Centre for Tobacco and Alcohol Studies, National Addiction Centre, Institute of Psychiatry, King's College London, London, UK

**Keywords:** Media, Advertising and Promotion, Public policy

## Abstract

**Aim:**

To examine the effects of tobacco control television advertisements with positive and negative emotional content on adult smoking prevalence and cigarette consumption.

**Design:**

Analysis of monthly cross-sectional surveys using generalised additive models.

**Setting:**

England.

**Participants:**

60 000 adults aged 18 years or over living in England and interviewed in the Opinions and Lifestyle Survey from 2004 to 2010.

**Measurements:**

Current smoking status, daily cigarette consumption, tobacco control gross rating points (GRPs—a measure of per capita advertising exposure), cigarette costliness, concurrent tobacco control policies, sociodemographic variables.

**Results:**

After adjusting for cigarette costliness, other tobacco control policies and individual characteristics, we found that a 400-point increase in positive emotive GRPs was associated with 7% lower odds of smoking (odds ratio (OR) 0.93, 95% CI 0.87 to 0.98) 1 month later and a similar increase in negative emotive GRPs was significantly associated with 4% lower odds of smoking (OR 0.96, 95% CI 0.92 to 0.999) 2 months later. An increase in negative emotive GRPs from 0 to 400 was also associated with a significant 3.3% (95% CI 1.1 to 5.6) decrease in average cigarette consumption. There was no evidence that the association between positive emotive GRPs and the outcomes differed depending on the intensity of negative emotive GRPs (and vice versa).

**Conclusions:**

This is the first study to explore the effects of campaigns with different types of emotive content on adult smoking prevalence and consumption. It suggests that both types of campaign (positive and negative) are effective in reducing smoking prevalence, whereas consumption among smokers was only affected by campaigns evoking negative emotions.

## Introduction

The effectiveness of tobacco control media campaigns has been extensively studied and there is strong empirical evidence that they can encourage quitting and reduce tobacco use among adults.^1–4^ Less is known, however, about the effectiveness of different message types on tobacco use, particularly the use of different emotional messages. Specifically, no study has looked at the role of the emotional content of television advertising in changing smoking behaviour using measures such as smoking prevalence and cigarette consumption. Rather, previous studies have focused on intermediate measures of effectiveness such as recall^5^ ^6^ or quit-based metrics such as calls to quit lines^7^ ^8^ and quit attempts^9–12^ which may not translate directly into successful quitting. For example, although caution is needed in directly applying research on marketing campaigns aimed to promote product use with those promoting smoking cessation, we note that recent research suggests that unprompted advertisement recall may underestimate the effectiveness of positive emotive brand campaigns and there are examples of brands that have had very effective campaigns but with low recall.^13^ ^14^

In England, tobacco control mass media campaigns were run consistently during the 2000s and studies have shown that the government-funded tobacco control television advertisements screened during this time period until April 2010 (when the government froze spending on national public health campaigns in England), were effective in increasing calls to quit lines^4^ and reducing smoking prevalence and cigarette consumption.^2^ The aim of this paper is to use the same television advertising data to explore the differential effectiveness of negative and positive emotional advertisements on adult tobacco use, specifically smoking prevalence and cigarette consumption.

## Methods

### Population survey data

The Opinions and Lifestyle Survey (OS), formerly the Office for National Statistics (ONS) Opinions Survey or ONS Omnibus Survey, is a monthly cross-sectional survey designed to be representative of adults living in private households throughout Great Britain.^15^ It is the main source of data used by the National Health Service Information Centre for studying smoking attitudes and behaviour and previous research shows that it provides reliable data on smoking.^16^ Over the study period, January 2004 to April 2010 inclusive, the number of months surveyed annually changed from a minimum of 9 months in 2004 to a maximum of 12 months in 2006 and 2008–2010 and response rates in each monthly sample varied from 58% to 69%. Each month, households are selected using a clustered, stratified multistage sample design and the interviewer carries out a face-to-face interview with one adult per household. Information is collected from respondents on a range of variables including age, gender, region of residence, employment status, education, gross income and smoking behaviour. We define a smoker as someone answering ‘Yes’ to the question ‘Do you smoke cigarettes nowadays?’. Based on two questions: ‘How many cigarettes a day do you usually smoke at weekends?’ and ‘How many cigarettes a day do you usually smoke on weekdays?’ which cover both manufactured cigarette and hand-rolled cigarette use, we derived an average number of cigarettes smoked a day (hereinafter termed average consumption). This was done by taking a weighted average of weekend (weight two-sevenths) and weekday (weight five-sevenths) consumption.

### Tobacco control policies

#### Tobacco control televised mass media campaigns

Film recordings of individual advertisements and measures of campaign exposure were obtained for government-funded televised tobacco control mass media campaigns in England from January 2004 to April 2010 from the Central Office of Information and the UK Department of Health Tobacco Marketing Team. Exposure to these campaigns was measured in gross rating points (GRPs). This is a standard measure of advertising exposure which reflects the average per-capita advertising exposure. For example, 400 GRPs/month, the minimum level that existing research suggests is associated with reductions in adult smoking prevalence,^1^ is equivalent to 100% of adults being exposed to four advertisements per month or 50% being exposed to eight advertisements. At an individual level, exposure will vary depending on frequency, channel and time of television viewing. For this study, adult GRPs for all tobacco control advertisements shown on television per month was used as the indicator of exposure to tobacco control television advertisements.

Using the film recordings obtained, we classified advertisements into three campaign types: (1) positive emotive campaigns if they evoked positive feelings about quitting (eg, pride, happiness, relief and satisfaction); (2) negative emotive campaigns if they evoked negative feelings about smoking (eg, worry, fear, disgust, guilt, anger, sadness); and (3) emotionally neutral campaigns (eg, campaigns designed to raise awareness of smoke-free legislation; see box 1). The advertisements were categorised independently by two researchers and we used a focus group comprising eight members of the UK Centre for Tobacco and Alcohol Studies’ Smokers Panel, run at the University of Bath, to validate both the framework and our coding. Further details about the coding framework and methodology can be found elsewhere.^17^

Box 1 **Details of positive and negative emotive campaigns**

Nearly all of the negative emotive adverts contained information about the negative consequences of smoking (mostly the direct health effects although a small number covered the health effects of second-hand smoke and some the emotional consequences of parental smoking on children). They were mostly testimonial or acted adverts with a very few showing graphic imagery. Positive campaigns focused on reasons for quitting and ways to quit and all were acted.

While both positive and negative emotive adverts provided basic information (either a phone number, website or text number that would lead to further information on quitting would appear on the screen), 94% of the positive emotive campaigns provided additional information on how to quit compared with just 3% of negative emotive advertisements.

Examples of and weblinks to adverts of each type are given in online supplementary table S1.

We obtained monthly GRP data for each advertisement shown during this period and, for each month, summed GRPs across all advertisements for each campaign type to derive a time series of monthly GRPs for each campaign type. During this period the Department of Health also funded Cancer Research UK and the British Heart Foundation to undertake media campaigns and we also include GRP data from these campaigns (all negative emotive campaigns). Together, these were the main purchasers of public sector tobacco control advertisements during this period.

#### Cigarette prices

We measured price using the weighted average retail selling price (WAP) of cigarettes (see online supplementary box S1), which more accurately reflects price trends than the most popular price category (MPPC),^18^ and in January 2011 replaced MPPC as the method for calculating tobacco excise levels in all European Union Member States.^19^ We then used WAP to derive a measure of cigarette costliness for each OS respondent (the ratio of WAP to self-reported monthly income in the month of interview). Monthly income represents the respondent's total gross income from all sources (earnings from employment and self-employment, pension, state benefits, interest from savings and other sources such as rent) before deductions for income tax, National Insurance, etc.

#### Other tobacco control policies

To quantify the degree of other tobacco control activity each month from 2004 until 2010 we used a coding scheme based on the Tobacco Control Scale (TCS) developed by Joossens and Raw 20 to compare tobacco control policies across Europe (see online supplementary table S2). The original TCS includes cigarette pricing and spending on public information campaigns which were excluded from our scheme as they were dealt with separately in our models (see above) using WAP and GRPs. Our scheme was therefore based on four policies (smoke-free work and public places, bans on advertising and promotion, health warning labels on cigarette packets and treatment to help smokers stop) and used a scoring system identical to the TCS.^20^

Information on the date and nature of policies implemented in England was obtained from an extensive literature search and informal discussions with tobacco control experts. Then for each month, scores for each policy were summed to derive a total Tobacco Control Score. Scores increased during the study period from 24.5 at the start, increasing to 27 in August 2005, 48 in July 2007 and 51 in January 2008 (see online supplementary figure S1).

### Design

The data set for analysis contained records for each adult aged 18 years and older living in England who was interviewed in the OS between January 2004 and April 2010 inclusive. Monthly data on GRPs (positive emotive, negative emotive and emotionally neutral), TCS and cigarette prices were merged with these data based on the month and year of the OS interview. Survey respondents with missing data on smoking status were excluded from both the smoking prevalence and cigarette consumption analyses (<0.07%). The analysis of cigarette consumption was further restricted to respondents who self-reported as being a current smoker in order to investigate whether consumption reduced among those who continued to smoke rather than in the population as a whole. Smokers with missing consumption data were also excluded from this analysis (<0.7%).

### Statistical analysis

We examined the association between GRPs and average cigarette consumption and smoking prevalence using Poisson generalised additive models (GAMs) and binomial logistic GAMs, respectively. GAMs allow us flexibility in the shape of the association between exposures and outcomes, specifically they allow us to fit smooth (non-linear) associations between exposures and outcomes, for example, regression spline functions fit piecewise (multiple joined) polynomial curves. As evidence suggests that tobacco control media campaigns have their effect on smoking behaviour while campaigns are being broadcast and for a short time afterwards,^2^ ^4^ ^21–23^ our initial models for each outcome included negative emotive GRPs and positive emotive GRPs during the month of interview (an immediate effect), and 1 and 2 months earlier (ie, lagged effects of 1 and 2 months) as three separate smooth terms. We also considered whether the impact of positive emotive advertisements on the outcomes varied with the intensity of negative emotive advertisements running simultaneously or in recently preceding months (and vice versa) by including an interaction between the smooth terms at the same lag and also at lags at which main effects of each type of advertisement were found. We also adjusted for other tobacco control television advertisements by including emotionally neutral GRPs during the month of interview, 1 and 2 months earlier as three separate smooth terms.

To adjust for the effect of other tobacco control policies implemented during the study period we included the TCS as a categorical term and individual cigarette costliness as a cubic regression spline to allow for a possible non-linear relationship (table 1). As monthly income reported by OS respondents was strongly skewed to the right, we log-transformed cigarette costliness before analysis to make the distribution more symmetrical. All models also included the following individual-level covariates potentially associated with smoking prevalence and consumption that may vary between OS surveys due to differential non-response cubic regression splines for age and income, and categorical variables for gender, government office region, education, employment status and social class (table 1). Number of adults per household was also included as a linear term to adjust for unequal probability of selection in the OS survey (only 1 adult per household is selected for interview); we could not adjust for the complex sampling design as the OS survey does not release survey design variables. Overdispersion was detected in the Poisson models and SDs were corrected using a quasi-Poisson model.

**Table 1:** Covariates in the generalised additive models

Covariates	Description
Tobacco control score	Categorical term for tobacco control score for England. 4 categories: 24.5, 27, 48 and 51
Cigarette costliness	Weighted average price of packet of 20 cigarettes in month of interview divided by average monthly gross income of respondent (ie, proportion of monthly income that a packet of cigarettes costs). Include as cubic regression spline
Number of adults in the household	Number of adults in households of OS respondent. Include as linear term
Age	Age of OS respondent. Include as cubic regression spline
Gender	Categorical term for gender of OS respondent
Government office region	Categorical term for government office region of residence of OS respondent. 9 categories: East Midlands, East of England, London, North East England, North West England, South East England, South West England, West Midlands and Yorkshire & the Humber
Social class	Categorical term for national statistics socioeconomic classification (NS-SEC) of OS respondent. 4 categories: managerial and professional occupations, intermediate occupations, routine and manual occupations, not classified
Employment	Categorical term for employment status of OS respondent. 3 categories: employed, unemployed, economically inactive
Education	Categorical term for highest level of qualification that OS respondent has received. 8 categories: degree level qualification (or equivalent), Higher educational qualification below degree level, A-Levels or Highers, ONC/National Level BTEC, O Level or GCSE equivalent (Grade A–C) or O Grade/CSE equivalent (Grade 1) or Standard Grade level 1–3, GCSE grade D-G or CSE grade 2–5 or Standard Grade level 4–6, Other qualifications (including foreign qualifications below degree level), No formal qualifications
Income	Total gross income from all sources before deductions for income tax, National Insurance etc. Include as a cubic regression spline

OS, Opinions and Lifestyle Survey.

The GAM Models were fitted in R.15.1.^24^ After adjustment for the covariates listed in table 1, GRP exposure variables were fitted in the model using cubic regression spline smooth terms. GRP exposure terms were removed from the model if they were not statistically significant (p>0.05) using backwards selection. The effective degrees of freedom (EDF) associated with each smooth term measures the degree of non-linearity, with an EDF of 1 indicating that the shape of the relationship between the term and the outcome, for example, log cigarette consumption, is linear. Smooth terms with an EDF equal to 1 were replaced with linear terms. The significance of interactions between GRP variables, for example between positive and negative emotive GRPs, was tested by using a variant of a tensor product smooth that allows interaction terms to be fitted in a model with smooth terms. We assessed the suitability of the final models, including whether there was any remaining temporal autocorrelation, that is correlation between adjacent terms in the time series, using plots of the residuals and carried out sensitivity analyses adjusting for time and seasonal effects, and found no evidence that our models were not appropriate. All tests were two-sided and performed at the 5% level of statistical significance. We report results in terms of changes in 400 GRPs per month, the current minimum level shown to be associated with reductions in adult smoking prevalence.^1^

## Results

The smoking prevalence and consumption analyses contained close to 60 000 and 14 000 survey respondents, respectively. Of the 26 222 GRPs during the study period, 53% were for negative emotive campaigns, 42% for positive emotive campaigns and the remaining 5% were emotionally neutral campaigns.

**Figure 1:**
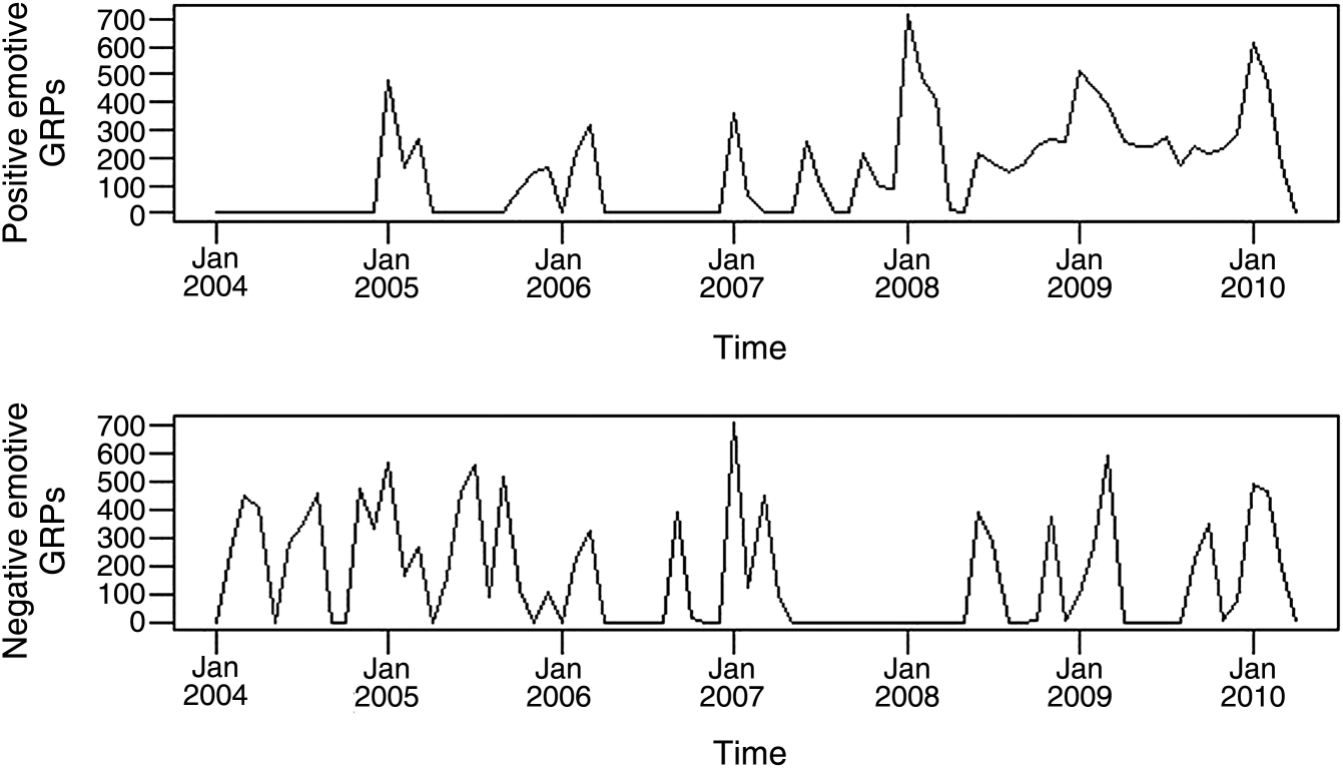
Time series plots of positive and negative emotive gross rating points.

The intensity of each campaign type changed during the study period, with more negative emotive campaigns in the earlier years and positive emotive campaigns in later years (figure 1). Adverts were screened in 60 of the 76 months of the study, with just negative emotive campaigns screened in 19 months, just positive emotive campaigns in 17 months and both in 24 months. In those months when negative emotive campaigns occurred, the median exposure was 281 GRPs (range 2.6–708 GRPs). In those months when positive emotive campaigns took place, the median exposure was 242 GRPs (range 14.6–718 GRPs). The correlation between negative and positive emotive campaigns during the study period was small (Pearson correlation coefficient=0.16, p=0.17). All interaction terms dropped out of both models during the backwards selection procedure.

After adjusting for cigarette costliness, other tobacco control policies and individual characteristics, an increase in positive emotive GRPs in the previous month and negative emotive GRPs in the previous 2 months was associated with a reduction in smoking prevalence (table 2). A 400-point increase in positive emotive GRPs was associated with 7% lower odds of smoking (0.93, 95% CI 0.87 to 0.98) 1 month later (lag 1) and a similar increase in negative emotive GRPs was associated with 4% lower odds of smoking (0.96, 95% CI 0.92 to 0.999) 2 months later (lag 2). We tested for an interaction between positive emotive GRPs at lag 1 and negative emotive GRPs at lag 2 but it was not significant (p>0.05).

**Table 2:** Results of Poisson and binomial logistic regression analyses to detect an association between positive and negative emotive GRPs and tobacco use

Outcome	Final models*	Linear term† OR (95% CI)	Smooth term EDF‡	p Value§
Average consumption	Negative emotive GRPs 1 month earlier		1.65	0.016
Smoking prevalence	Positive emotive GRPs 1 month earlier	0.93 (0.87 to 0.98)		0.01
	Negative emotive GRPs 2 months earlier	0.96 (0.92 to 0.999)		0.04

*All regression models were adjusted for cigarette costliness, tobacco control score, number of adults in the household, gender, age, income, social class, education, employment status and government office region of residence. GRPs for each campaign type at different lags were initially considered as smooth terms and we used backwards selection of the GRPs to find the best model. Any GRP term in the final model found to be linear (EDF=1) was replaced with a linear term.

†ORs and 95% CIs reported for smoking prevalence associated with a 400-point increase in GRPs.

‡The effective degrees of freedom (EDF) is a measure of how ‘wiggly’ the term is (ie, EDF=1 corresponds to a straight line, ie, a linear effect).

§p Value from a t test on the parametric regression coefficients and F test on smooth terms.

EDF, effective degrees of freedom.

**Figure 2:**
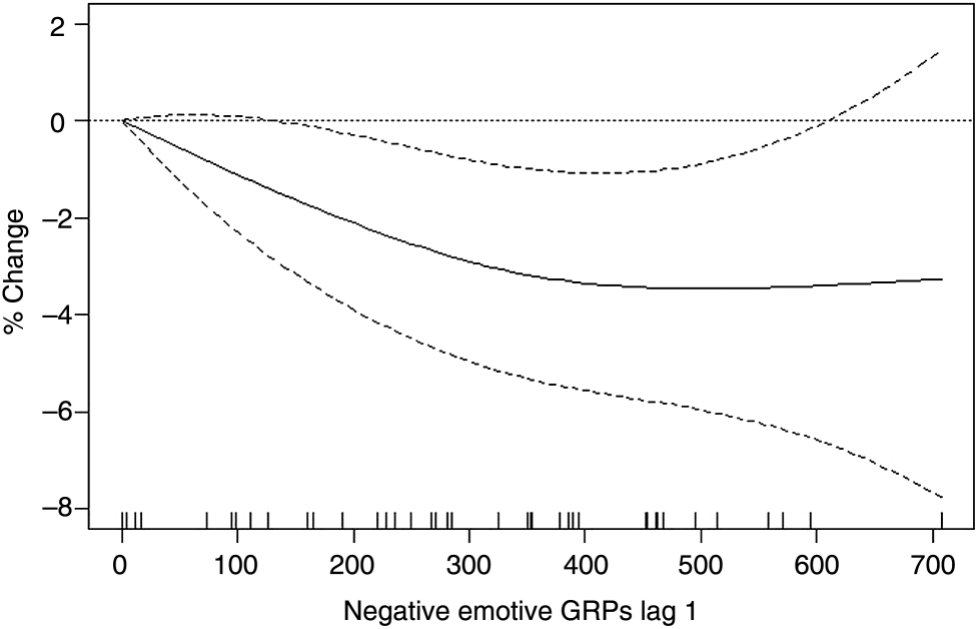
Estimated effect of negative emotive gross rating points (GRPs) at lag 1 on average consumption (solid lines) and 95% CIs (dashed lines). The y-axis shows the % change in consumption for different values of these GRPs compared to a baseline value of 0. The rug plot along the bottom of the graph depicts the each observation.

In contrast to smoking prevalence, cigarette consumption among smokers was negatively associated with only negative emotive GRPs in the previous month. To interpret the smooth term for negative emotive GRPs in the previous month (table 2), figure 2 shows the per cent change in consumption for different values of GRPs compared to having 0 GRPs. For example, a change in GRPs from 0 to 400 was associated with a 3.3% (95% CI 1.1% to 5.6%) decrease in average consumption 1 month later. The graph indicates that compared to 0 GRPs, the per cent reduction in average consumption gets larger for increasing in GRPs until GRPs reach around 400, after which the per cent change remains fairly constant.

## Discussion

We found that both positive and negative emotive campaigns are effective in reducing tobacco use. After adjusting for cigarette costliness, other tobacco control policies and smoker characteristics, a 400-point increase in positive emotive GRPs was significantly associated with 7% lower odds of smoking (OR=0.93) 1 month later and a similar increase in negative emotive GRPs was significantly associated with 4% lower odds of smoking (OR=0.96) 2 months later. Only increases in negative emotive GRPs were associated with reductions in consumption among smokers; an increase in GRPs from 0 to 400 was associated with a significant 3.3% decrease in cigarette consumption. Finally, we identified a dose–response between negative emotive GRPs and consumption which increased up to 400 GRPs and then levelled off. This is consistent with previous research that gives a recommended level of 400 GRPs per month (1200 per quarter).^1^ ^25^ This has important implications: (1) if campaign exposure does not reach 400 GRPs each month it may not be maximally effective; and (2) paying for more than 400 GRPs in other months may not add any additional benefit in terms of reducing consumption among smokers. There is therefore scope for realising greater efficiency which, given funding constraints, is important.

One limitation of our study was that, although we adjusted for other known major determinants of tobacco use (cigarette costliness and other tobacco control policies, sociodemographic characteristics) and are unaware of any other determinant of tobacco use linked with both GRPs and our tobacco use metrics, we cannot completely rule out that other, unmeasured variables may confound the relationship between GRPs and smoking outcomes. We adjusted for the effect of seasonality in a sensitivity analysis, but it was not statistically significant. The OS survey does not release survey design variables and we were unable to adjust SEs for the complex multistage design involving clustering and stratification. We also used population-level data for the WAP of manufactured cigarettes, whereas in reality: (1) individuals vary widely in their brand choice and price trends have varied markedly by brand^18^ and (2) 25% of the OS respondents smoked exclusively or mainly hand-rolled cigarettes for which we did not have price data. The emotionally neutral GRPs also included GRPs from two campaigns (‘Small Steps’ and ‘Tips’) for which campaign creatives were not available. The two campaigns combined represent 68.2 GRPs during the period studied. However, given that between January 2004 and April 2010 there were, on average, 3800 tobacco control GRPs per year, these represent a small proportion of all GRPs.

Nevertheless, the study has a number of important strengths including the use of population level smoking behaviour outcome data, the investigation of non-linear relationships and interactions, and the control for a large number of potential confounders including other tobacco control measures. The study's key advantage over the existing literature is that it was able to benefit from the variability in type of mass media campaign screened in the UK in being the first study to examine the effectiveness of different types (positive and negative emotive) antismoking mass media campaigns on population smoking prevalence and consumption. Previous studies evaluating different campaign types generally used proxy measures of impact (eg, recall or calls to quit lines),^5–8^ ^10^ ^12^ while those evaluating impacts of mass media campaigns on prevalence and consumption have been based in jurisdictions (largely Australia) where campaigns have been almost exclusively negative.^1^ This has led to conclusions that negative emotive campaigns are most effective.^1^ A key finding of our paper, therefore, is that positive campaigns were also effective in reducing smoking prevalence, the difference in impact on prevalence of the two campaign types being statistically indistinguishable. Importantly, although our study found that the effects of positive emotive campaigns were seen at a lag of 1 month, and that of negative emotive campaigns at a lag of 2 months, there was no statistical evidence for interaction to suggest that the effects of positive campaigns were being driven by previous negative campaigns.

Our finding of the impact of positive campaigns may be explained in part by difficulties in accurately disentangling the various different effective elements of adverts and accurately coding these in studies undertaken in different jurisdictions.^1^ ^26^ For example, predominantly negative emotive adverts may still have positive elements which could, in part, drive their effectiveness. It is also possible that the nature of the advert (eg, testimonial content) rather than its emotive content determines its success. A recent US study found that adverts that were highly emotional, personal testimonial or both were more effective than other adverts.^27^ This group of effective adverts includes some that would have been coded as positive adverts in our study as they involved personal stories of the quitting process and quitting strategies. One Australian study has shown that screening a negative health effects advertisement followed by an advertisement that illustrated the benefits of the quit line was more efficient in generating quit line calls than negative health effects advertisements alone.^7^ The general literature also shows that fear appeals in public health campaigns are most effective when they also have a message to enhance efficacy.^28^ ^29^ Efficacy in tobacco control could be conceived as an individual's belief as to whether (1) the strategy proposed (eg, a quit kit) will enable quitting and (2) he/she is able to use the strategy proposed to quit smoking. While all the advertisements we coded (both positive and negative emotive) provided basic information (either a phone number, website or text number that would lead to further information on quitting would appear on the screen), 94% of the positive emotive campaigns provided additional information on how to quit and therefore a strategy aimed to enhance efficacy, compared with just 3% of negative emotive advertisements. It is possible that this combination of negative and positive content is key to campaign effectiveness.^7^ ^28^ ^29^

In conclusion, exposure to both positive and negative emotive advertisements was associated with reductions in smoking prevalence and exposure to negative emotive advertisements was associated with reductions in cigarette consumption among smokers. Further population-based studies should examine the impact of different campaign types and content, particularly the role of positive messaging, on smoking prevalence and quitting. Future work could also benefit from experimental studies to explore further how best to optimise the effectiveness of these messages, for example, whether and how different message types are best combined or sequenced, and the optimum time lag if sequenced. The ideal would be to work with campaign planners to screen different campaign types at varying intervals and evaluate the impacts contemporaneously.

**What this paper adds**

- To date, no study has explored the role of emotional content in television advertisements in reducing smoking prevalence and cigarette consumption.
- This observational study of adult tobacco use found that both positive and negative emotive advertisements were associated with reductions smoking prevalence whereas cigarette consumption in smokers was only affected by campaigns evoking negative emotions.

## Supplementary Material

Web supplement
