# Supplementary material for: Effectiveness of tobacco control television advertisements with different types of emotional content on tobacco use in England, 2004–2010
Source: Tob Control. 2014 Jul 18;25(1):21–6. doi: 10.1136/tobaccocontrol-2013-051454 (PMC4717366; doi:10.1136/tobaccocontrol-2013-051454)
Supplement: Web supplement [file tobaccocontrol-2013-051454-s1.pdf]

SUPPLEMENTARY MATERIAL

Effectiveness of tobacco control television advertisements with different types of emotional content on tobacco use in England, 2004-2010

Sims, M., Langley, T., Lewis, S., Richardson, S., Szatkowski, L., McNeill, A., Gilmore, A.B.

Table S1. Examples of positive emotive, negative emotive and emotionally neutral advertisements shown in England 2004-2010.

Campaign	Emotional content	Advertisement description
Emotional Consequences (Anthony Hicks)[1]	Negative	Features Anthony Hicks, a 58-year-old smoker with throat and lung cancer, lying in a hospital bed struggling to breathe. Mr Hicks talks about his illness and how his daughter is due to visit him. The following image says that he died 10 days after filming and never got to see his daughter again.
Give up before you clog up [2]	Negative	A group of friends in a pub are disgusted to see the fatty deposits caused by smoking come out of their cigarettes. Advertisement also shows an artery with fatty deposits caused by smoking to show the damage cigarettes were doing.
Reasons[3]	Positive	Featured a number of adults talking about their reasons for giving up smoking. Advertisement showed parents talking about all the things they're looking forward to doing with their children. These included weddings; teaching their kids to drive; special holidays; and holding their grandchildren.
Right tools for the job (Umbrella) [4]	Positive	Focuses on the idea that whatever you're trying to achieve, it is much easier when you have the right tools. Advertisement launched the Quit Kit as the right tool to help smokers quit. Features a man using a cocktail umbrella to keep himself dry on a rainy day. It then shows the man getting home to find his Quit Kit on the doormat, demonstrating that you need to right tools to succeed. The

		advertisement then shows the man using the right tool: a full-size umbrella.
Countdown [5]	Neutral	Legislation came into force banning smoking in public places in England on 1 July 2007. The campaign was devised to ensure everybody affected by the law knew how it would affect them. Features an 'everyday' man walking through a variety of locations including a cafe, pub, garage and office – all of which have become smokefree environments.

1 NHS. Emotional Consequences. 2004. Available at: http://smokefree.nhs.uk/resources/news/campaigns/2004-smokefree-campaign-testimonials-anthony-hicks/ (accessed 21 October 2013). (Archived by WebCite® at http://www.webcitation.org/6Ilkixkq6 on 22 July 2013)

2. NHS & British Heart Foundation. Give up before you clog up. 2004. Available at: http://smokefree.nhs.uk/resources/news/campaigns/give-up-before-you-clog-up/ (accessed 21 October 2013). (Archived by WebCite® at http://www.webcitation.org/6KYV66NqD on 22 October 2013)

3. NHS. Reasons. 2008. Available at: http://smokefree.nhs.uk/resources/news/campaigns/2008-smokefree-campaign-reasons/ (accessed 21 October 2013). (Archived by WebCite® at http://www.webcitation.org/6KYVNSZwY on 22 October 2013)

4. NHS. Right Tools for the Job. 2010. Available at: http://smokefree.nhs.uk/resources/news/campaigns/2010-smokefree-campaign-right-tools-for-the-job-umbrella/ (accessed 21 October 2013). (Archived by WebCite® at http://www.webcitation.org/6KYVCiS5R on 22 October 2013)

5. NHS. Countdown.2008. Available at: http://smokefree.nhs.uk/resources/news/campaigns/2007-smokefree-campaign-countdown/ (accessed 21 October 2013). (Archived by WebCite® at http://www.webcitation.org/6KYUtHHYc on 22 October 2013)

Table S2. Coding scheme for tobacco control policies (a shortened version of the Tobacco Control Scale in Europe[1])

**Smoke free work and other public places**	**22**
**Workplaces excluding cafes and restaurants – one only of**	**10**
Complete ban without exceptions (no smoking rooms); enforced	10
Complete ban, but with closed, ventilated, designated smoking rooms; enforced	8
Complete ban, but with ventilated, designated smoking rooms; enforced	6
Meaningful restrictions; enforced	4
Legislation, but not enforced	2
**Cafes and restaurants – one only of**	**8**
Complete ban; enforced	8
Complete ban, but with closed, ventilated, designated smoking rooms ; enforced	6
Meaningful restrictions; enforced	4
Legislation, but not enforced	2
**Public transport and other public places – additive**	**4**
Complete ban in domestic trains without exceptions	1
Complete ban in other public transport without exceptions	1
Complete ban in educational, health, government and cultural places without exceptions	2
OR Ban in educational, health, government and cultural places, but with designated smoking areas or rooms	1
**Comprehensive bans on advertising and promotion (points for each type of ban - additive)**	**13**
Complete ban on tobacco advertising on television	3
Complete ban on outdoor advertising (eg. posters)	2
Complete ban on advertising in print media (eg. newspapers and magazines)	2
Complete ban on indirect advertising (eg. cigarette branded clothes, watches, etc)	2
Ban on point of sale advertising	1
Ban on cinema advertising	1
Ban on sponsorship (0.5 for domestic and 0.5 for international)	1

Ban on internet advertising	0.5
Ban on radio advertising	0.5
**Large direct health warning labels**	**10**
**Rotating health warnings**	**2**
**Size of warning – one only of**	**4**
10% or less of packet	1
11 – 25% of packet	2
26 – 40% of packet	3
41% or more of packet	4
**Contrasting colour (eg. black lettering on white background)**	**1**
**Pictorial health warnings**	**3**
**Treatment to help dependent smokers stop**	**10**
**Quitline – one only of**	**2**
Well funded national quitline or well funded quitlines in all major regions of country	2
OR National quitline with limited funding or a patch work of small local quitlines	1
**Network of smoking cessation support (3) and reimbursement of treatment (3)**	**6**
Cessation support network covering whole country (3); free (3)	6
Cessation support network, but only in selected areas, eg. major cities (2); free (3)	5
Cessation support network covering whole country (3), partially free (2)	5
Cessation support network, but very limited, just a few centres (1), free (3)	4
Cessation support network, but only in selected areas, eg. major cities (2), partially free (2)	4
Cessation support network covering whole country (3), not free (0)	3
Cessation support network, but very limited, just a few centres (1), partially free (2)	3
Cessation support network, but only in selected areas, eg. major cities (2); not free (0)	2
Cessation support network, just a few centres (1), not free (0)	1
**Reimbursement of medications – one only of**	**2**
Reimbursement of pharmaceutical treatment products	2

OR Partial reimbursement of pharmaceutical treatment products	1
**MAXIMUM POSSIBLE SCORE**	**55**

[1] Joossens L, Raw M. The Tobacco Control Scale: a new scale to measure country activity. *Tobacco Control* 2006;**15**:247-53.

Figure S1 Tobacco Control Score for England, January 2004- April 2010

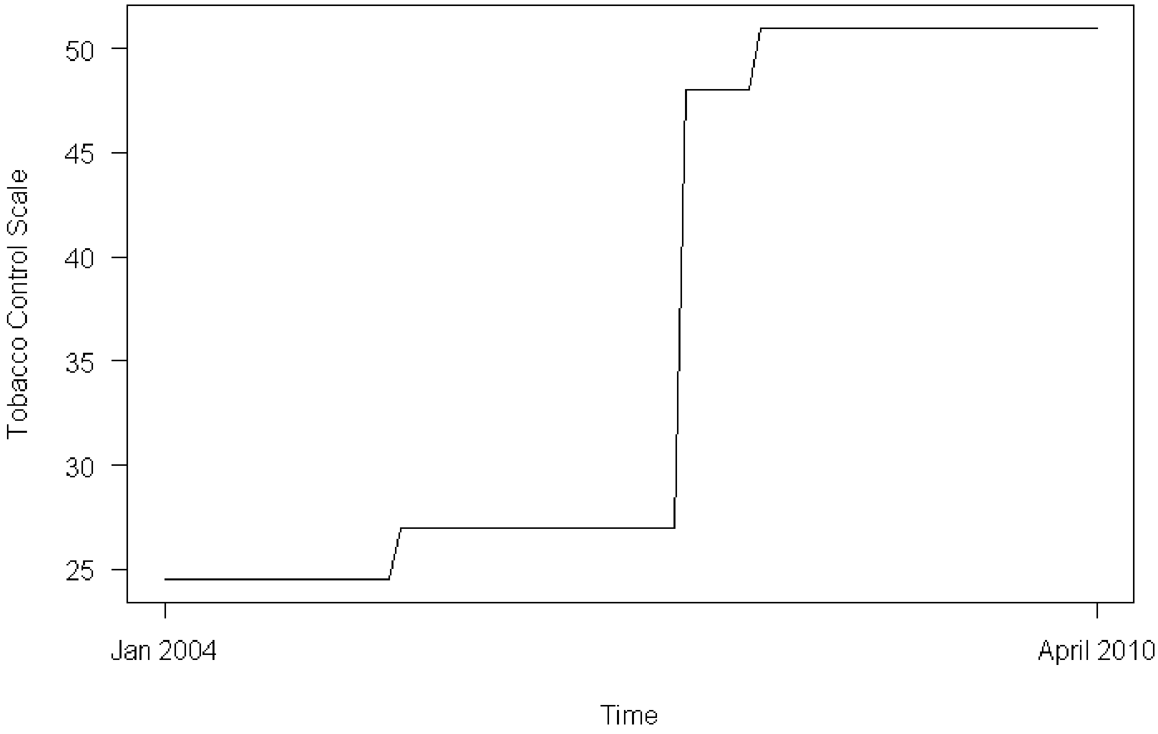

Box S1. Weighted average price of cigarettes

We calculated weighted average price as:

$$\sum_{i=1}^{n} m_i p_i \qquad \text{(Eqn 1)}$$

where p_i is the price of cigarette brand *i*, m_i is the volume market share of cigarette brand *i* and n is the number of cigarette brands. Cigarette price data were obtained for packs of 20 cigarettes (or 19 cigarettes for brands sold only in 19s). There is no single source of price data available for the whole study period. From 2002 to 2005, we obtained recommended retail prices of all major cigarette brands for the UK market from PriceChecker, a supplement of the weekly magazine 'Retail Newsagent' published in the UK. We used June data (published in May) and December data (published in November) from PriceChecker, except in 2005 when October data were used (PriceChecker ceased publication in October 2005). From November 2006, Nielsen began to publish cigarette sales data, including price and volume (the number of cigarette packs sold for each brand), for the Great Britain market. We obtained 7 monthly data between November 2006 and May 2008 (thus including November 2006, May and November 2007 and May 2008) and monthly data from November 2008 to December 2011. The expense of Nielsen data prevented us obtaining monthly data before November 2008. A comparison between the two data sources indicated that PriceChecker and Nielsen could provide comparable price data over time.[2]

Volume market share was obtained from the General Household Survey (GHS) for 2002 to 2005 and Nielsen from 2006 to 2010 (as detailed above). GHS, an annual survey designed to be representative of the population of Great Britain, asks smokers aged 16 years and over which brand of cigarettes they smoke and how many sticks they smoke a week. These questions were used to calculate market share by brand.

An exploratory analysis of trends in WAPs from November 2008 until December 2011 (the time period when monthly data were available) indicated it was reasonable to derive a WAP for months when cigarette price and volume were unavailable by linearly interpolation.

[1] Council Directive 2011/64/EU of 21 June 2011 on the structure and rates of excise duty applied to manufactured tobacco. Available at: http://eur-lex.europa.eu/LexUriServ/LexUriServ.do?uri=OJ:L:2011:176:0024:0036:EN:PDF (last accessed 18th November 2013).

[2] Gilmore AB, Tavakoly B, Taylor G, Reed H. Understanding tobacco industry pricing strategy and whether it undermines tobacco tax policy: the example of the UK cigarette market. Addiction. 2013;108:1317-26.
